# Molecular and Antiangiogenic Effects of Paclitaxel-Loaded Nanoparticles: Influence of the Nanocarrier Type

**DOI:** 10.1021/acs.molpharmaceut.5c01740

**Published:** 2026-03-06

**Authors:** Julia Sapienza Passos, Giovanna B. de Melo, Giovanna C. Salata, João Agostinho Machado-Neto, Alyssa Panitch, Luciana B. Lopes

**Affiliations:** † Department of Pharmacology, Institute of Biomedical Sciences, 54544 University of Sao Paulo, Sao Paulo, Sao Paulo 05508-000, Brazil; ‡ Wallace H. Coulter Department of Biomedical Engineering, 1372 Georgia Institute of Technology and Emory University, Atlanta, Georgia 30332, United States

**Keywords:** breast cancer, paclitaxel, nanostructured lipid carriers, hybrid nanoparticles, pharmacodynamics

## Abstract

Nanostructured lipid carriers (NLCs) and lipid–polymeric hybrid nanoparticles (H-NPs) were developed for the local administration of paclitaxel (PTX) and breast cancer therapy. Here, we investigated how nanoparticle type and composition influence the molecular effects and in vivo antiangiogenic activity of PTX. Elevated BAX expression and PARP-1 cleavage in MCF-7 and MDA-MB-231 breast cancer cells treated with nanoencapsulated PTX indicate that apoptosis is the primary mechanism of cell death, regardless of the nanocarrier type. However, distinct molecular effects were observed for other markers. Both unloaded nanocarriers increased α-tubulin acetylation in MCF-7 cells, indicating an intrinsic ability of the carriers to modulate cytoskeletal organization. Upon PTX loading, these effects became carrier-dependent: NLC-PTX induced higher α-tubulin acetylation than H-NP-PTX compared to the PTX solution. Moreover, in MCF-7 cells, NLC-PTX, but not H-NP-PTX, markedly enhanced drug-induced DNA damage, increasing γH2AX expression by 13.4-fold compared to PTX as a solution. These findings suggest that the nanocarriers not only act as delivery systems but may also confer additional biological effects that may contribute to PTX cytotoxicity. In the chicken chorioallantoic membrane model, nanoencapsulation reduced PTX-induced irritation from moderately irritant (irritation score 6) to nonirritant while preserving its antiangiogenic activity, achieving a 6.1–7.8-fold inhibition of vessel growth at subcytotoxic doses. Collectively, these results highlight nanoencapsulation as a promising strategy to potentiate PTX activity while improving safety for local breast cancer therapy. The distinct molecular responses of lipid and hybrid systems demonstrate that nanocarrier composition and structure modulate biological outcomes, underscoring the importance of rational nanocarrier design to overcome current therapeutic challenges.

## Graphical abstract

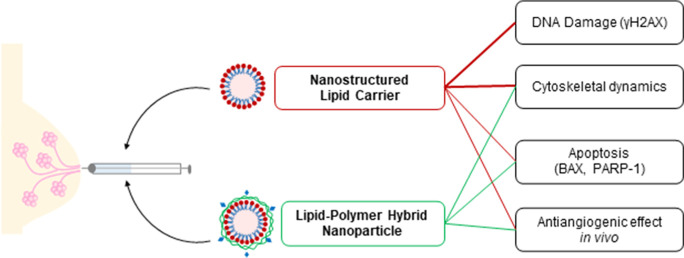

## 1 Introduction

Breast cancer is the most commonly diagnosed cancer and the leading cause of cancer-related death among women worldwide.^1^ Despite the recent advances in early detection and development of new therapeutic agents, multidrug resistance and tumor recurrence still represent major clinical challenges.^2^ Paclitaxel (PTX), a microtubule-stabilizing agent, is frequently employed as the first-line chemotherapeutic drug in breast cancer treatment.^1^ It binds to the β-tubulin subunit of microtubules, promoting their stabilization and preventing depolymerization.^3^ The increased microtubule stability leads to cell cycle arrest in the late G2/M phase, ultimately inducing cell death and inhibiting cellular replication.

In addition to its well-characterized cytotoxic activity via tubulin binding, PTX also exerts other important molecular effects that contribute to its in vivo therapeutic efficacy. PTX activates multiple signal transduction pathways, some of which are associated with pro-apoptotic signaling. For example, it can trigger the activation of mitogen-activated protein kinases (MAPKs), leading to the phosphorylation of the antiapoptotic protein BCL-2 and the dephosphorylation of pro-apoptotic proteins BAX and BAD.^4^ Furthermore, PTX has been shown to induce apoptosis through the activation of caspase-3 and subsequent cleavage of poly­(ADP-ribose) polymerase (PARP-1).^5^ In addition, its antiangiogenic properties arise from both direct cytotoxicity toward endothelial cells via microtubule disruption, G2/M arrest, increased BAX/BCL-2 ratio, and PARP-1 cleavage as well as from the downregulation of key angiogenic mediators such as vascular endothelial growth factor (VEGF), thrombospondin-1 (TSP-1), and angiopoietin-1 (Ang-1).^6^

In spite of its multiple mechanisms of action, paclitaxel’s clinical use is difficult because of its poor aqueous solubility and toxic profile. The conventional commercial formulation, which was introduced in the clinics multiple years after the drug was discovered, requires solubilization in Cremophor EL (polyoxyethylated castor oil) and ethanol (1:1, w/w), a vehicle associated with severe adverse effects including hypersensitivity reactions and peripheral neuropathy.^7^ These limitations have driven the development of several drug delivery systems for paclitaxel, with nanoparticle-based drug delivery systems emerging as a promising alternative, including the albumin-bond formulation of paclitaxel Abraxane.^8^ By improving drug solubility and stability, enabling modified release, and enhancing cellular uptake, nanoparticles can significantly alter the pharmacokinetic profile and adverse effects of encapsulated drugs.^9,10^

Less understood is the interaction of nanoparticles with cellular components. These interactions depend on nanoparticles’ chemical composition and surface properties, which in turn affect nanoparticle–cell interactions, uptake and fate, and drug molecular effects. Consequently, the pharmacodynamics and therapeutic outcome of the encapsulated drug change with “identity” and surface properties.^11^ Many times, the nanoparticle per se exerts cellular effects independently of the drug,^12^ which imparts new pharmacodynamic effects to the combo nanoparticle-drug.

We have previously demonstrated the relevance of nanoparticle composition and functionalization to its route of internalization, drug release, and cytotoxicity.^13^ We developed nanostructured lipid carriers (NLCs) and hybrid nanoparticles (H-NPs), the latter composed of the NLC as a core surrounded by a shell of the thermoresponsive polymer poly­(*N*-isopropylacrylamide) (PNIPAM) functionalized with the type I collagen-binding peptide SILY, for the intraductal delivery of paclitaxel.^13,14^ The hybrid nanoparticle was designed to combine the advantage of a lipid core, which enables paclitaxel solubilization, with a thermoresponsive shell that modulates drug release at physiological temperature and allows functionalization with SILY, a peptide that binds to collagen type I (an extracellular matrix protein overexpressed in the mammary tumor microenvironment) to improve breast ductal retention and selectivity.^13^ These characteristics make the hybrid nanoparticle particularly suitable for intraductal administration, an innovative route that still faces technical limitations, such as the difficulty of cannulation.^15^ By extending the residence time of the drug within the ducts, SILY-modified nanoparticles can help reduce the frequency of administration, complications associated with cannulation, and patient discomfort and improve adherence to treatment. Hybrid nanoparticles exhibited an average diameter of approximately 380.0 ± 4.4 nm, PDI of 0.28 ± 0.05, and zeta potential of −15.7 ± 2.0 mV, with a paclitaxel encapsulation efficiency of 68.3 ± 1.3%.^13^ In contrast, NLCs showed a smaller average diameter (233.4 ± 7.2 nm), lower PDI (0.18 ± 0.03), zeta potential of −11.9 ± 0.5 mV, and a higher encapsulation efficiency (92.9 ± 0.3%).^13,14^ While the NLC was internalized by clathrin- and caveolin-mediated endocytosis, the hybrid nanoparticles seemed to enter cells exclusively via clathrin-mediated endocytosis. We also observed a lower IC_50_ of NLCs compared to paclitaxel solution and the hybrid nanoparticle in breast cancer spheroids.^13^ Building upon these studies, we hypothesized that differences in nanoparticle composition, structure, and surface functionalization, as well as biological effects triggered by the nanocarriers per se, might have an influence on the molecular effects of paclitaxel. The present study was designed to test this hypothesis.

Using cultures of breast cancer cells with varying receptor expression profiles, we investigated whether the nanoparticle type influences the cytoskeleton organization, DNA damage, protein expression, and cell death pathways involved in the molecular signaling of PTX. Because of the importance of PTX antiangiogenic effects for chemotherapy, we also assessed whether its nanoencapsulation hindered PTX efficacy to reduce VEGF-induced angiogenesis in a chorioallantoic membrane model. With this study, we aimed to contribute to the overall understanding of the influence of nanoencapsulation on the drug pharmacodynamic effects.

## 2 Experimental Section

### 2.1 Materials

Paclitaxel was purchased from Cayman Chemical (Ann Arbor, MI, USA). Polysorbate 80, tributyrin, PBS (phosphate-buffered saline), DMSO (dimethyl sulfoxide), phenylmethylsulfonyl fluoride (PMSF), sodium orthovanadate (Na_3_VO_4_), tetrasodium pyrophosphate (Na_4_P_2_O_7_), sodium dodecyl sulfate (SDS, 10% w/v in water), 2-acrylamido-2-methyl-1-propanesulfonic acid (AMPS), potassium persulfate (KPS), and *N*,*N*-*bis*(acryloyl)­cystamine (BAC) were acquired from Sigma-Aldrich (St. Louis, MO, USA). Acrylic acid (AAc) was purchased from Thermo Fisher Scientific (Waltham, MA, USA), and ethylenediaminetetraacetic acid (EDTA) and NaF (sodium fluoride) were purchased from Synth. Soy phosphatidylcholine (PC) was purchased from Avanti Polar Lipids (Alabaster, AL, USA), and glyceryl behenate (Compritol 888 ATO) was kindly supplied by Gattefosse (Saint-Priest, France). Hydrazide-modified collagen-binding peptide RRANAALKAGELYKSILYGSG-hydrazide (SILY-hydrazide, molecular weight 2252.6 kDa, 80% purity) was purchased from Innopep (San Diego, CA, USA), and poly­(*N*-Isopropylacrylamide) (PNIPAM) was acquired from Polysciences Inc. (Warrington, PA, USA). PNIPAM and BAC were stored under nitrogen at 4 °C. Other specific reagents (such as the ones employed in cell culture and Western blot) are described along with the respective methodology. Ultrapure water was used unless stated in the individual methods.

### 2.2 Nanoparticle Obtainment

Paclitaxel-loaded and unloaded nanostructured lipid carriers (NLCs) and hybrid nanoparticles (H-NPs) were prepared as previously reported.^13,14^ Briefly, to obtain the NLCs, the heated oil phase (tributyrin, Compritol 888 ATO, soy phosphatidylcholine at 3.5:3.5:3, w/w/w, 10% of the NLC content) was mixed with the aqueous phase (PBS containing 3% of polysorbate 80, w/v). The final mixture was then immersed in a water bath for temperature control and probe sonicated for 20 min (50 s on and 30 s off) and 40% amplitude (VCX500, Sonics, Newtown, CT). Paclitaxel-loaded NLCs (NLC-PTX) were prepared by adding the drug to the oil phase before the inclusion of the aqueous phase (final concentration of 1%, w/w of the formulation).

Hybrid nanoparticles were obtained by fabricating a PNIPAM shell around the NLC (as the core) using a precipitation polymerization reaction;^13^ for paclitaxel-loaded hybrid nanoparticles (H-NP-PTX), the NLC-PTX was used as the core. As a general procedure, the NLCs were diluted at 1:10 (v/v) in Milli-Q water, added to a three-neck round-bottom flask, and heated at 60 °C under nitrogen for 20 min. Then, PNIPAM, AMPS, SDS, AAc, BAC, and KPS were added for polymerization, which was allowed to proceed for 4 h. SILY-modified hybrid nanoparticles were synthesized using EDC/NHS activation, as previously described.^13^ Briefly, 20 mg of H-NPs were dispersed at 5 mg/mL in a buffer containing 0.1 M MES, 8 M urea, 10 mM EDC, and 20 mM NHS (pH 4.5) and incubated for 30 min to activate surface functional groups. Following activation, 12.8 mg of SILY peptide was added and allowed to react for 90 min. After the reaction, the samples were filtered using tangential flow filtration to remove unreacted reagents and excess peptide, frozen, lyophilized, and stored at room temperature.

### 2.3 Influence of the Nanoparticle Type on the Molecular Effects of PTX

#### 2.3.1 Cell Culture

Breast cancer cells (MCF-7 and MDA-MB-231) were obtained from ATCC (Manassas, VA, USA). MCF-7 cells represent a more common breast cancer phenotype (luminal A, positive for estrogen, ER, and progesterone receptors, PR), while MDA-MB-231 is a model of triple-negative breast cancer (negative for ER, PR, and HER2). The cells were cultured in Dulbecco’s Modified Eagle Medium:Nutrient Mixture F12 (DMEM-F12) culture medium supplemented with 10% fetal bovine serum (FBS, Gibco/Invitrogen, USA) and 1% penicillin/streptomycin. The cells were maintained at 37 °C under 5% CO_2_ and 95% humidity and passaged when the cells reached 80% confluency.

#### 2.3.2 Assessment of the Expression of Proteins Involved in Cytoskeleton Stability, DNA Damage, and Apoptosis

Western blot was employed to assess whether the type of nanoparticle (NLCs and H-NPs) influences the PTX mechanism of action and molecular effects, including DNA damage, protein expression, and cell death pathways.

MCF-7 and MDA-MB-231 cells were seeded (5 × 10^5^ cells/plate) in 100 mm Petri dishes and incubated at 37 °C and 5% CO_2_ for 24 h. Subsequently, cells were treated for 48 h. The experimental groups were: (i) negative control (nontreated), (ii) positive control (paclitaxel solution, 1% w/w in propylene glycol), (iii) NLC, (iv) NLC-PTX, (v) H-NP, and (vi) H-NP-PTX. Cells were treated using IC_50_, IC_50_/2, and 2 × IC_50_ concentrations determined in previous studies of our group after cell treatment for a longer period of time (72 h).^13,14^ This experimental design was used to ensure that, especially at higher doses (2 × IC_50_), a large fraction of cells was still viable at the time of molecular analysis, minimizing confounding effects from widespread cell death while capturing relevant alterations in protein expression levels. This methodology acknowledges the time-dependent nature of drug responses and is consistent with established practice where viability end points and mechanistic end points are optimized at different time points to best capture their respective biological phenomena.^16^

After treatment, total protein was extracted using a lysis buffer containing 100 mM *Tris* (pH 7.5), 1% Triton X-100, 2 mM PMSF, 10 mM Na_3_VO_4_, 100 mM NaF, 10 mM Na_4_P_2_O_7_, and 4 mM EDTA.^17^ The protein content in each sample was quantified using the BCA protein assay kit (Sigma-Aldrich, St. Louis, MA, USA), following the manufacturer’s instructions. Equal amounts of protein (30 μg per sample) were separated by SDS-PAGE and transferred onto nitrocellulose membranes by electrophoresis. Membranes were blocked with 5% nonfat dry milk in TBS-T (10 mM Tris, 150 mM NaCl, and 0.05% Tween 20, w/v) and incubated with specific primary antibodies diluted in 3% nonfat dry milk in TBS-T, followed by incubation with HRP-conjugated secondary antibodies. Detection was performed using the Pierce ECL Western Blotting substrate (ThermoFisher, MA, USA) and a G:BOX Chemi XX6 documentation system (Syngene, Cambridge, UK). All membranes were processed and imaged under equivalent experimental and exposure conditions, and multiple exposure times were evaluated during image acquisition. The bands’ intensities were measured using ImageJ software. Antibodies used and their respective details are listed in Table S1.

#### 2.3.3 Evaluation of Cytoskeletal Alterations

An immunofluorescence assay was employed to assess morphological and cytoskeletal alterations induced by the nanoparticles.^18^ MCF-7 and MDA-MB-231 cells were seeded in 12-well plates at 3 × 10^4^ cells/well, with a slide coverslip placed at the bottom of each well. After 24 h of incubation, cells were treated with paclitaxel (either in solution or incorporated into NLC or H-NP) or with unloaded-nanoparticles at IC_50_/2 concentration for 48 h. After treatment, coverslips were washed twice with PBS, fixed with 4% paraformaldehyde for 15 min, permeabilized with 0.1% Triton X-100 for 10 min, and blocked with 1% BSA in PBS for 30 min. Cells were incubated with AlexaFluor 488-conjugated anti-α-tubulin monoclonal antibody (clone DM1A, eBioscience, Thermo Scientific, IL, USA) at 5 μg/mL in 0.1% BSA for 1 h in the dark. Nuclear staining was performed using Fluoroshield with DAPI (Abcam, MA, USA), and coverslips were mounted onto glass slides. Images were acquired using a Lionheart FX automated microscope (BioTek, Agilent, VT, USA).

### 2.4 Influence of the Nanoparticle Type on the Antiangiogenic Effects of PTX

The chorioallantoic membrane (CAM) of eggs was used to evaluate the antiangiogenic potential and differences between paclitaxel-loaded and unloaded-NLCs and H-NPs. The protocol was conducted in accordance with the guidelines from the Brazilian Council for Control of Animal Experimentation (CONCEA) and approved by the Animal Care and Use Committee (IACUC) at the Institute of Biomedical Sciences of the University of São Paulo (CEUA-ICB-USP, no. #1950170124).

#### 2.4.1 Assessment of Irritant Potential—Hen’s Egg Test—Chorioallantoic Membrane (HET-CAM) Assay

Before the antiangiogenic effects were assessed, it was necessary to ensure that PTX concentrations associated with antiangiogenic effects were safe in the CAM model. For this objective, fertilized Leghorn chicken eggs (#02B/HW408, Globoaves, SP, Brazil) were incubated at 37 ± 0.5 °C and 70% humidity for 9 days with automatic rotation every 2 h.^19^ On the day of the experiment, the chorioallantoic membrane (CAM) was exposed by using a rotary saw blade, and 100 μL of each treatment was carefully applied. The experimental groups were as follows: (i) 0.9% NaCl (negative control), (ii) 0.1 M NaOH (positive control), (iii) PTX solution (1% w/w in propylene glycol, which is the content added to the nanoparticles), (iv) PTX solution diluted in 0.9% NaCl (final drug concentration 10 nM, which was shown to inhibit angiogenesis^20^), (v) propylene glycol (vehicle control for PTX solution), (vi) unloaded NLC, (vii) NLC-PTX, (viii) unloaded H-NP, and (ix) H-NP-PTX.

Each treatment was performed in 3 eggs, and the development of hemorrhage, vascular lysis, and coagulation was evaluated within 30 s to 5 min after application. The chorioallantoic membranes were photographed before, during, and after treatment to demonstrate the changes. For NLC and NLC-PTX, which have a milky aspect, post-treatment images were taken after the membrane was rinsed with 1 mL of 0.9% NaCl. The irritation index was calculated based on the onset times of vascular responses using the following equation as previously described:^21^

$$Score=\left(\left(\frac{301-t_{h}}{300}\right)\times 5\right)+\left(\left(\frac{301-t_{l}}{300}\right)\times 7\right)+\left(\left(\frac{301-t_{c}}{300}\right)\times 9\right)$$

Where *t*_h_, *t*_l_, and *t*_c_ represent the time (in seconds) of the first appearance of blood hemorrhages, vessel lysis, and coagulation, respectively. Scores were interpreted as nonirritating (0–0.9), slightly irritating (1–4.9), moderately irritating (5–8.9), or severely irritating (9–21).^21^

#### 2.4.2 Antiangiogenic Assay on Chorioallantoic Membrane

To assess paclitaxel’s antiangiogenic activity, CAM assays were performed using paclitaxel formulations diluted in 0.9% NaCl to obtain final concentrations of 10 nM PTX, which was found to be safe in the CAM model. VEGF (500 nM) was used to induce angiogenesis, aiming to promote robust and reproducible vascular network formation within short experimental time frames.^22–24^ In preliminary studies, we observed that increasing VEGF concentrations resulted in progressively stronger vascular responses, allowing the selection of experimental conditions that generated a robust and quantifiable angiogenic network within the evaluated time window. When assessing antiangiogenic effects in combination with paclitaxel, we selected a 500 nM VEGF concentration to establish a robust vascular network within 48 h, thereby enabling more reliable detection of treatment-induced vascular inhibition. Importantly, although expressed as a nanomolar concentration, this condition corresponds to an absolute amount of ∼1.9 μg of VEGF per CAM. Comparable VEGF doses in the high nanogram to microgram range (e.g., 0.5–4 μg per CAM) have been reported in CAM studies to induce angiogenesis within 24–48 h, supporting our choice.^22,23^

Fertilized Leghorn chicken eggs (#02B/HW408, Globoaves, SP, Brazil) were incubated at 37 ± 0.5 °C and 70% humidity for 6 days with automatic rotation every 2 h.^24,25^ On day 6, the CAM was exposed and photographed (initial assay time: *t*_initial_). The experimental groups were as follows: (i) 0.9% NaCl (negative control), (ii) 500 nM VEGF, (iii) 10 nM paclitaxel solution +500 nM VEGF, (iv) NLC-PTX (diluted in 0.9% NaCl to a final concentration of 10 nM of paclitaxel) +500 nM VEGF, (v) NLC (diluted in 0.9% NaCl using the same ratio as NLC-PTX) +500 nM VEGF, (vi) H-NP-PTX (diluted in 0.9% NaCl to a final concentration of 10 nM of paclitaxel) +500 nM VEGF, (vii) H-NP (diluted in 0.9% NaCl using the same ratio as H-NP-PTX) +500 nM VEGF. Each treatment (100 μL) was applied carefully to the CAM of 3–4 eggs, and eggs were sealed with Parafilm and adhesive tape to prevent contamination and dehydration. After 48 h of incubation at 37 ± 0.5 °C and 70% humidity (without rotation), the parafilm was removed, and new images were obtained (*t*_final_).

Images were converted to grayscale using the software ImageJ (Washington, DC, USA) and analyzed as previously described.^12^ Briefly, circular areas of 5 cm diameter were selected in the images before (*t*_initial_) and after (*t*_final_) determination of the mean pixel value for each image. The mean pixel value was used to calculate the relative vessel growth (RVG) using the following equation:

$$RVG\left(\%\right)=\left(\frac{P_{i}-P_{f}}{P_{f}}\right)\times 100$$

where *P*_i_ and *P*_f_ represent the average pixel intensity at the initial and final assay time, respectively.

### 2.5 Statistical Analysis

All data are expressed as mean ± standard deviation from at least three independent experiments. Statistical analyses were performed using GraphPad Prism 8 software (San Diego, CA, USA). The data was analyzed for differences using *t* tests for comparisons between two groups. For multiple comparisons, ANOVA was employed with a Tukey post hoc test. Differences were considered significant when *p* < 0.05.

## 3 Results

### 3.1 Lipid and Hybrid Nanoparticles Display Distinct Effects Depending on the Cell Type

To evaluate the impact of paclitaxel nanoencapsulation and nanoparticle type on the drug’s pharmacodynamic properties, we first analyzed the expression of proteins involved in cytoskeleton stability, DNA damage, and apoptosis. α-Tubulin was selected as the loading control since, although paclitaxel targets microtubules, its primary effect is on tubulin dynamics and polymerization status rather than on total tubulin protein levels. Accordingly, α-tubulin expression remains stable under paclitaxel treatment under our experimental conditions, making it suitable for use as a loading control. Importantly, the Western blot analysis was performed under denaturing conditions, which reflect total α-tubulin content rather than its polymerized or dynamic state.

Treatment-mediated changes in protein expression were dependent on the type of nanoparticle and cell line. Acetyl-α-tubulin was assessed as a marker of microtubule stability directly related to the mechanism of action of paclitaxel.^26^ The nanocarrier type influenced tubulin acetylation in a manner that was dependent on the cell line. In MCF-7 cells, Sol-PTX, NLC-PTX, and H-NP-PTX increased acetyl-α-tubulin expression compared to the untreated cells by up to 4.9-, 9.7-, and 2.3-fold, respectively (Figure 1A–C). Compared to the PTX solution, the most pronounced effect was mediated by the NLC-PTX (2.4-fold), while the effect of the PTX-loaded H-NP was inferior to that of the drug solution at all concentrations tested. Curiously, acetyl-α-tubulin expression increased upon cell treatment with unloaded NLC and H-NP (at both IC_50_ and 2 × IC_50_) compared to untreated cells, suggesting that the nanocarriers (and/or their components) interfere with cytoskeletal dynamics. In MDA-MB-231 cells (Figure 1B–D), the increases in acetyl-α-tubulin expression induced by the NLC-PTX and PTX solutions were comparable, while the H-NP-PTX-mediated effect was less pronounced. In addition, unloaded NLC, but not H-NP, increased acetyl-α-tubulin expression in MDA-MB-231 cells. Together, these results suggest that, if we compare the effects of PTX-loaded nanocarriers with PTX solution in both cells lines, more pronounced tubulin acetylation resulted from NLC-PTX treatment.

**Figure 1:**
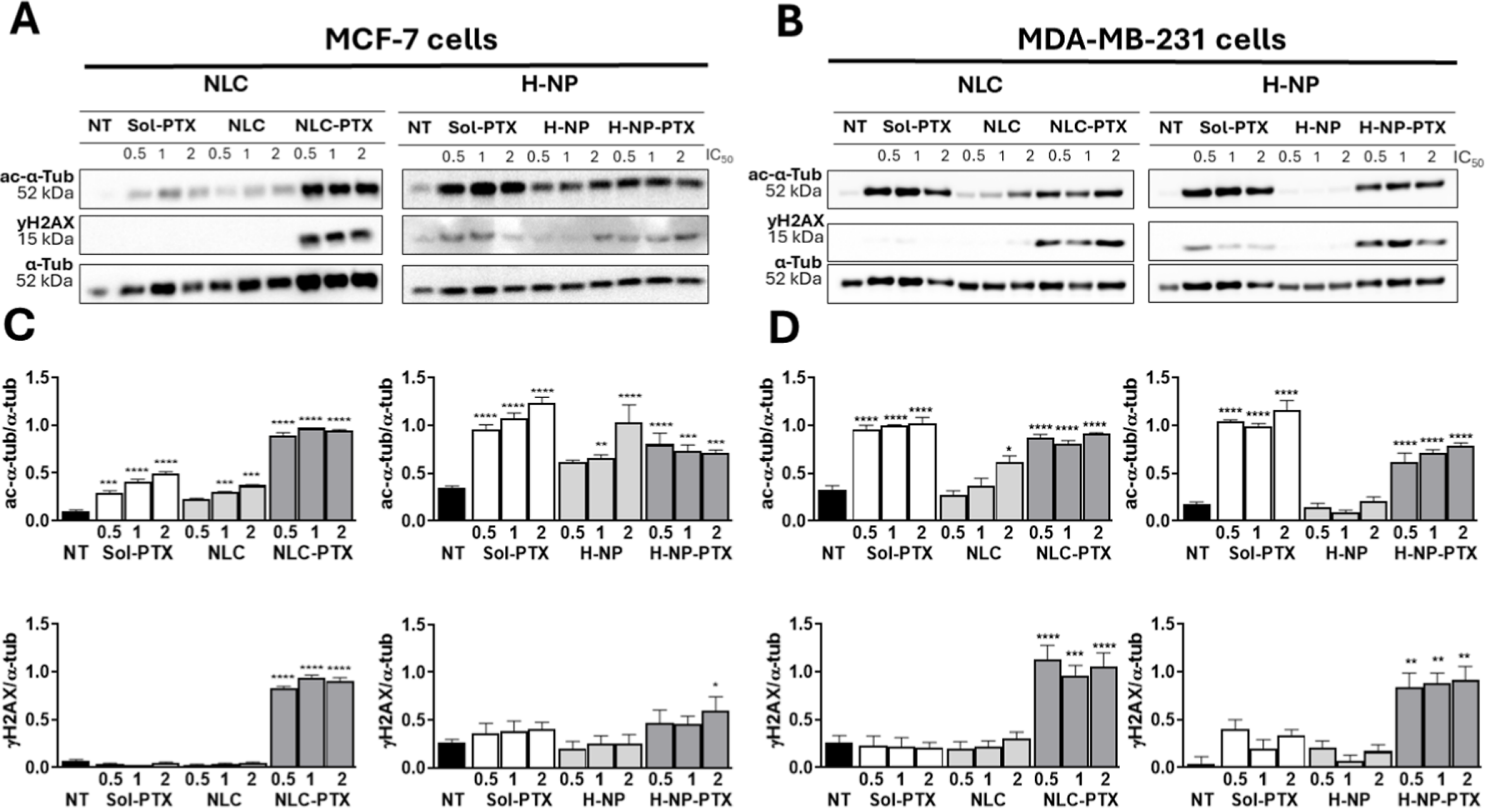
Expression of γH2AX and acetylated alpha-tubulin (ac-α-tub) proteins in MDA-MB-231 and MCF-7 breast tumor cells treated with unloaded or paclitaxel-loaded nanoparticles and a drug solution for 48 h; α-tubulin was used as a loading control. (A) Representative images of bands obtained by Western blotting in MCF-7 cells, (B) representative images of bands obtained by Western blotting in MDA-MB-231 cells, (C) expression of γH2AX and ac-α-tub proteins in MCF-7 cells, and (D) expression of γH2AX and ac-α-tub proteins in MDA-MB-231 cells. Cells were treated for 48 h using concentrations corresponding to IC_50_/2, IC_50_, and 2 × IC_50_ values previously determined from 72 h monolayer cytotoxicity assays. NT: untreated cells (control), Sol-PTX: paclitaxel in propylene glycol, NLC: nanostructured lipid carrier, NLC-PTX: paclitaxel-loaded NLC, H-NP: hybrid nanoparticle, and H-NP-PTX: paclitaxel-loaded H-NP.

Next, we analyzed the expression of γH_2_AX, a key marker of DNA damage.^27^ MCF-7 cells treated with paclitaxel-loaded NLCs exhibited the highest γH2AX expression levels (3.7–13.4-fold compared to untreated control), indicating that NLC-PTX induced a markedly stronger DNA damage response compared to PTX-solution and H-NP-PTX under the conditions tested. No substantial change in γH2AX expression was observed in cells treated with unloaded nanoparticles. These findings suggest that NLCs not only act as drug carriers but may also enhance paclitaxel-induced DNA damage, thereby contributing to increased cytotoxicity in MCF-7 cells. This observation is consistent with the lower IC_50_ observed for NLC-PTX compared with paclitaxel solution and H-NP-PTX in MCF-7 cells.^13,14^ In MDA-MB-231 cells, both PTX-loaded nanocarriers displayed significant effects, further highlighting cell-type-dependent responses, with triple-negative cells showing greater susceptibility to H-NP-PTX.

We also evaluated the expression of BAX, a key pro-apoptotic protein.^28^ BAX expression was increased in both cells treated with NLC-PTX and H-NP-PTX (Figure 2). Compared to the PTX solution, hybrid nanoparticles induced substantial increases in BAX expression (6.3-fold) in MCF-7 cells, while the NLC effect was more pronounced in MDA-MB-231 cells (4.6-fold). These results support a relationship between cell type/receptor status and nanoparticle-mediated PTX effects.

**Figure 2:**
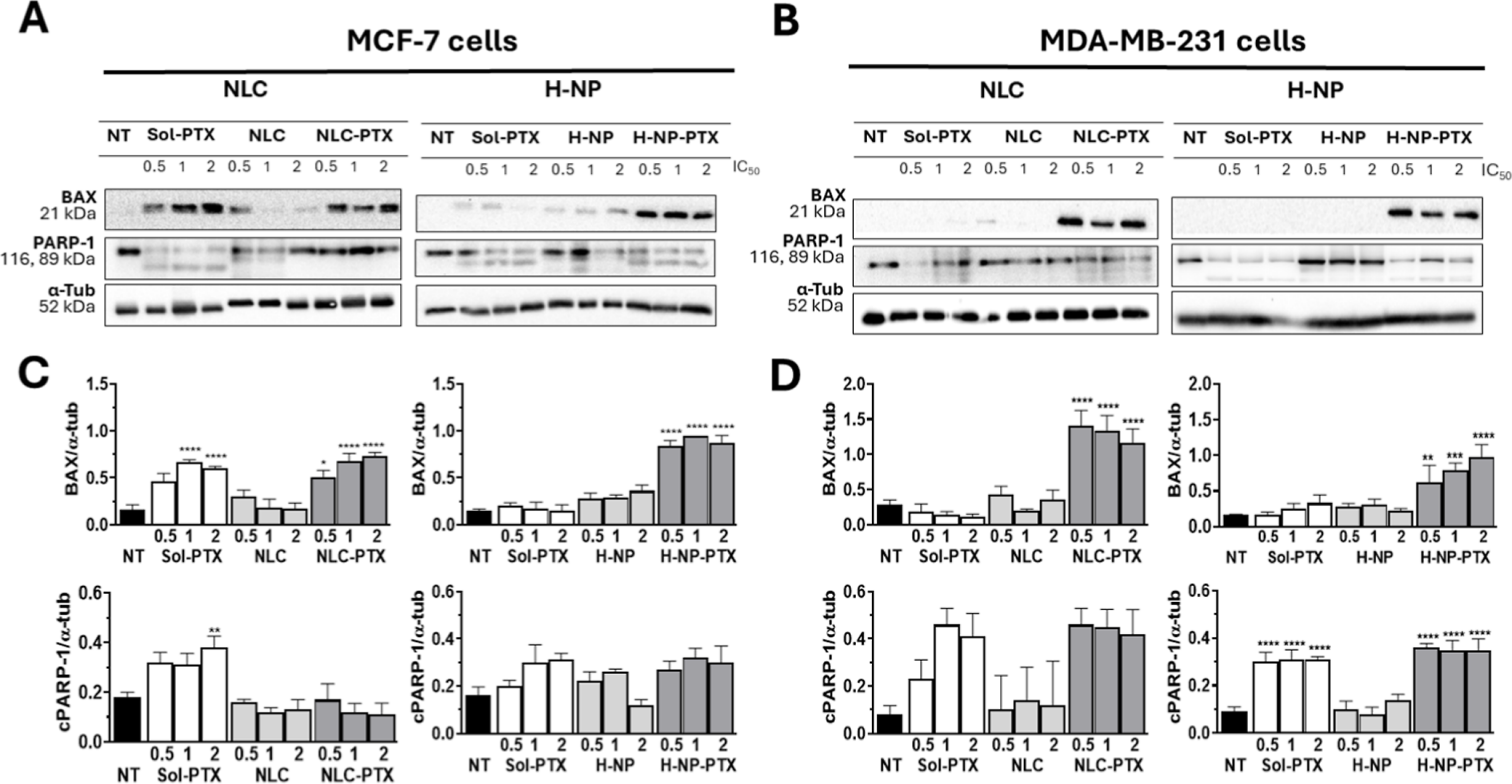
Expression of proteins related to cell death mechanisms, BAX and PARP-1 (cleaved portion, cPARP-1) proteins, in MDA-MB-231 and MCF-7 breast tumor cells treated with unloaded or paclitaxel-loaded nanoparticles and a drug solution for 48 h; α-tubulin was used as a loading control. (A) Representative images of bands obtained by Western blotting in MCF-7 cells, (B) representative images of bands obtained by Western blotting in MDA-MB-231 cells, (C) expression of BAX and cPARP-1 proteins in MCF-7 cells, and (D) expression of BAX and cPARP-1 proteins in MDA-MB-231 cells. Cells were treated for 48 h using concentrations corresponding to IC_50_/2, IC_50_, and 2 × IC_50_ values previously determined from 72 h monolayer cytotoxicity assays. NT: untreated cells (control), Sol-PTX: paclitaxel in propylene glycol, NLC: nanostructured lipid carrier, NLC-PTX: paclitaxel-loaded NLC, H-NP: hybrid nanoparticle, and H-NP-PTX: paclitaxel-loaded H-NP.

Finally, we examined PARP-1 cleavage (cPARP-1), a hallmark of apoptotic pathway activation (Figure 2).^29^ PARP-1 cleavage was observed in cells treated with paclitaxel, without significant differences between drug solution and nanoparticles, confirming that paclitaxel-induced cell death occurs predominantly through apoptosis, independent of its encapsulation.

### 3.2 Loaded and Unloaded Nanoparticles Cause Cytoskeleton Changes

Given that Western blot analysis revealed significant alterations in acetylated-tubulin expression following treatment with PTX-loaded and unloaded nanoparticles, we performed an immunofluorescence assay to further investigate the impact of these treatments on microtubule organization, as well as nuclear and cellular morphology (Figures 3 and 4).

**Figure 3:**
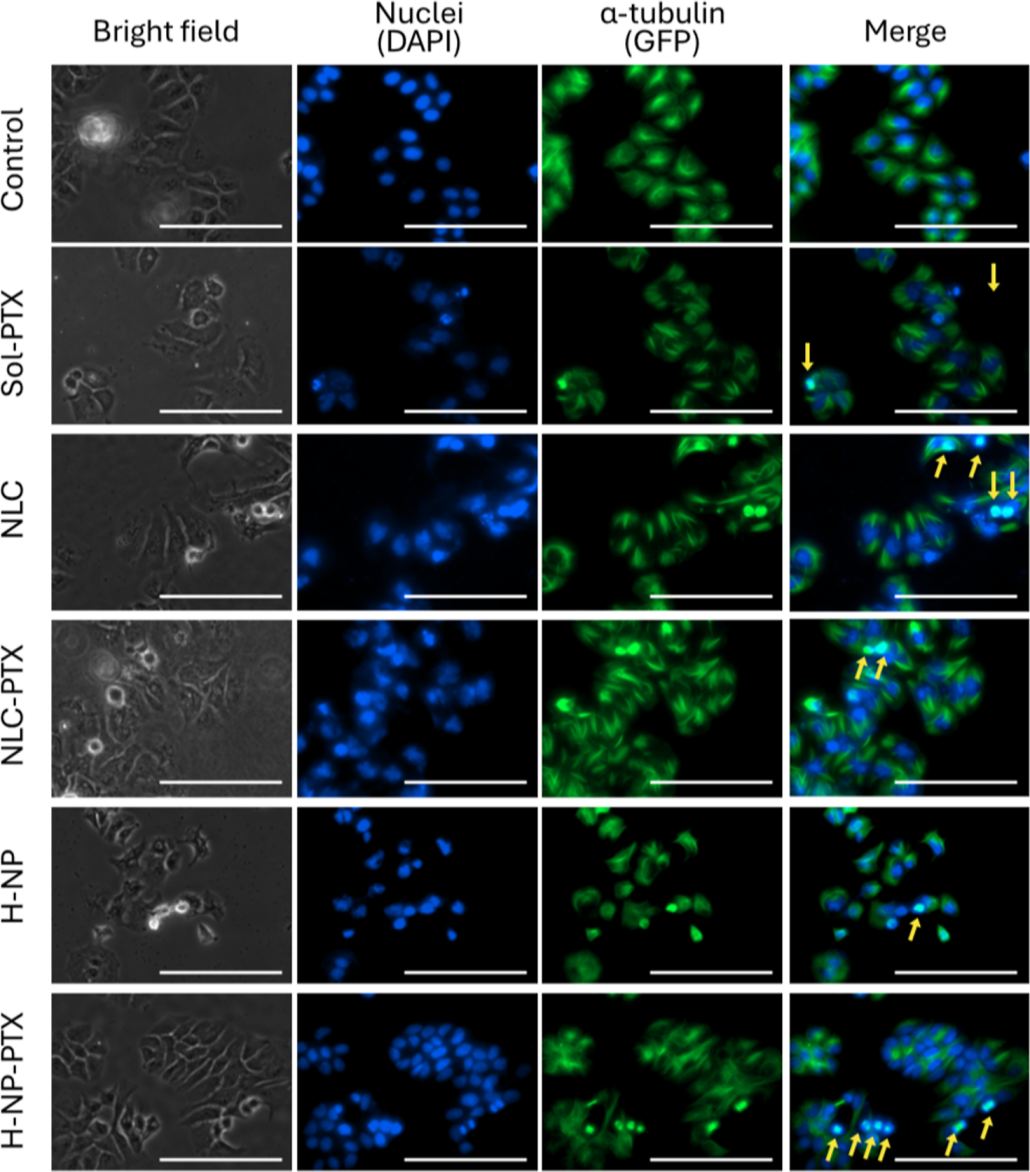
Cytoskeleton evaluation of breast cancer MCF-7 cells by immunofluorescence. The brightfield image shows cell morphology, DAPI stains nuclei (blue), and Alexa Fluor 488-conjugated anti-α-tubulin antibody highlights microtubules forming the cytoskeleton (GFP—green). Images were obtained from cells treated for 48 h with paclitaxel solution (Sol-PTX), nanostructured lipid carrier (NLC), paclitaxel-loaded NLC (NLC-PTX), hybrid nanoparticle (H-NP), and paclitaxel-loaded H-NP (H-NP-PTX) at IC_50_/2 values. Yellow arrows indicate the overlap between tubulin and the cell nucleus. Scale bar = 100 μm.

**Figure 4:**
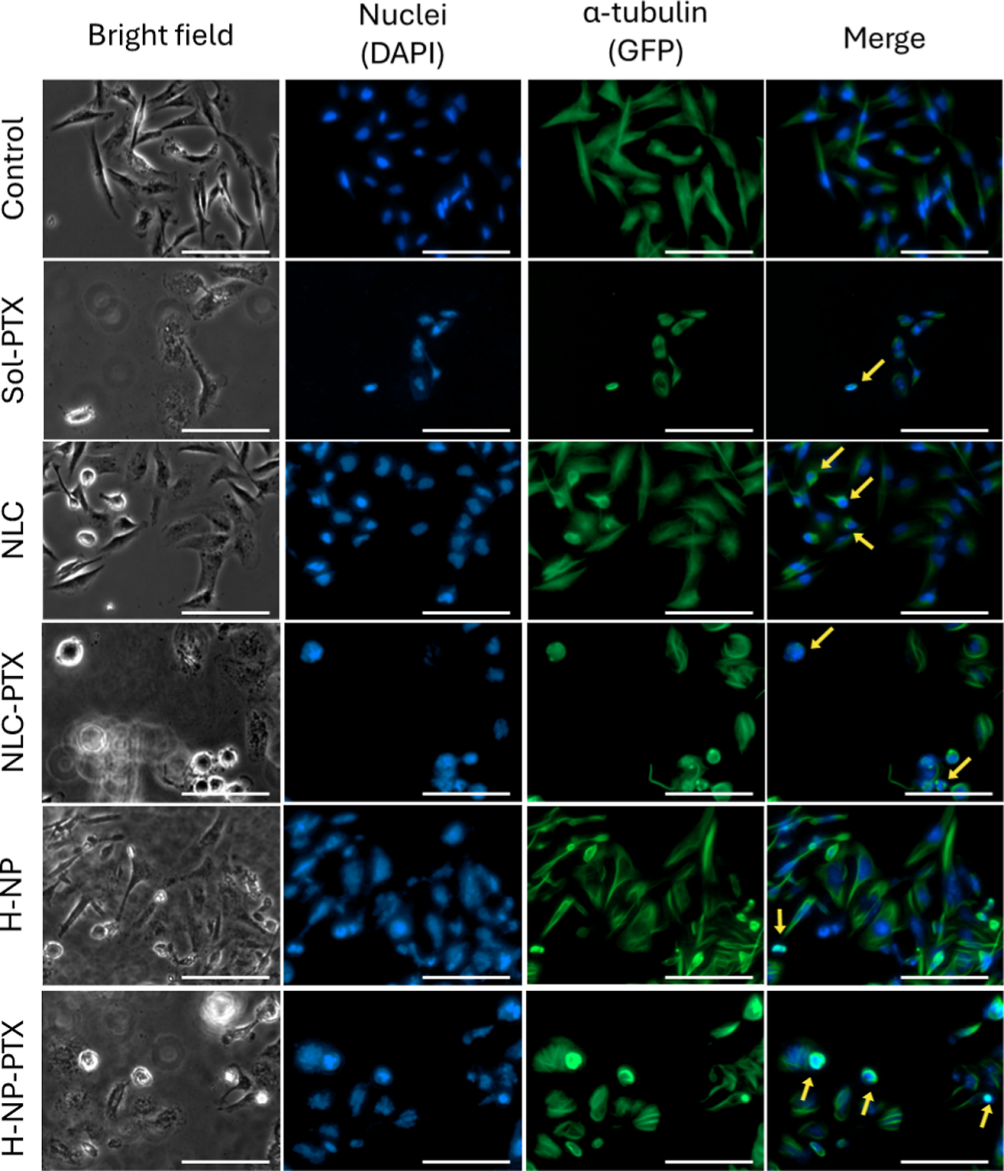
Influence of treatment on the morphology and cytoskeleton of breast cancer MDA-MB-231 cells by immunofluorescence. The brightfield image shows cell morphology, DAPI stains nuclei (blue), and Alexa Fluor 488-conjugated anti-α-tubulin antibody highlights microtubules forming the cytoskeleton (GFP—green). Images were obtained from cells treated for 48 h with paclitaxel solution (Sol-PTX), nanostructured lipid carrier (NLC), paclitaxel-loaded NLC (NLC-PTX), hybrid nanoparticle (H-NP), and paclitaxel-loaded H-NP (H-NP-PTX) at IC_50_/2 values. Yellow arrows indicate the overlap between tubulin and the cell nucleus. Scale bar = 100 μm.

In the untreated control group, a higher number of cells was observed. In MCF-7 cells, a slightly rounded morphology was evident (Figure 3), whereas MDA-MB-231 cells displayed a more elongated shape (Figure 4), which is consistent with the characteristic and expected morphologies of these cell lines. In both cases, microtubules were homogeneously distributed throughout the cytoplasm, surrounding the nuclei, which is consistent with cells in a proliferative state and with preserved microtubule architecture.

A reduced number of cells was observed after treatment with paclitaxel solution (Sol-PTX), particularly in MDA-MB-231 (Figure 4), along with marked cytoskeletal disruption and cells undergoing mitosis, as evidenced by the presence of mitotic spindles. In bright-field images, morphological alterations were apparent, and detached and/or dead cells were also observed.

Treatment with unloaded NLCs also induced cytoskeletal alterations. Cells exhibiting prominent mitotic spindles and multinucleation were detected, suggesting mitotic arrest. These alterations were more pronounced in MCF-7 cells, in agreement with the changes in acetylated-tubulin expression detected by Western blot. Notably, cells displaying almost complete overlap of DAPI (nuclei) and GFP (tubulin) signals were identified, potentially indicating mitotic catastrophe (a condition in which cells initiate but fail to complete mitosis, leading to microtubule collapse onto the nucleus, as indicated by yellow arrows).^30^ These observations help to justify the cytotoxicity of unloaded NLCs.^13,14^ Similar results were obtained for cells treated with NLC-PTX, although more intense spindle staining was observed, suggesting an enhanced microtubule-stabilizing effect of this formulation, most likely due to paclitaxel’s microtubule depolymerization-inhibiting activity. Such alterations were also observed in the MDA-MB-231 cells.

Cells exposed to H-NP-PTX exhibited morphological and fluorescence features consistent with cytoskeletal disruption and mitotic catastrophe, indicating that nanoencapsulation preserves paclitaxel’s ability to interfere with microtubule organization even in more complex nanostructured systems.

### 3.3 PTX Nanoencapsulation Protects CAM from PTX Irritative Effects

Before the antiangiogenic effects were assessed, it was necessary to ensure that PTX concentrations associated with these effects were safe in the CAM model. The HET-CAM assay was used to assess the irritation potential of lipid and hybrid nanoparticles by evaluating vascular alterations (hemorrhage, lysis, and coagulation) over 5 min of treatment (Figure 5). While saline caused no visible changes to the chorioallantoic membrane, treatment with 0.1 M NaOH induced vascular lysis and hemorrhage, resulting in an irritation score of 18 (severely irritating). The control paclitaxel solution (1% w/w in propylene glycol, similar to the concentration used in the nanoformulations) caused some hemorrhage and coagulation points with an irritation score of 6 (moderately irritating). This effect was attributed to the drug as the vehicle alone (propylene glycol) caused no visible vascular changes. Membrane treatment with paclitaxel-loaded nanoparticles, regardless of the type, caused no vascular alterations (irritation score of zero), demonstrating that the developed nanoparticles can protect the chorioallantoic membrane from the vascular irritation observed with a higher concentration of the free drug. Similarly, unloaded nanoparticles did not cause vascular toxicity, suggesting that regardless of the type, they can be considered safe for local administration.

**Figure 5:**
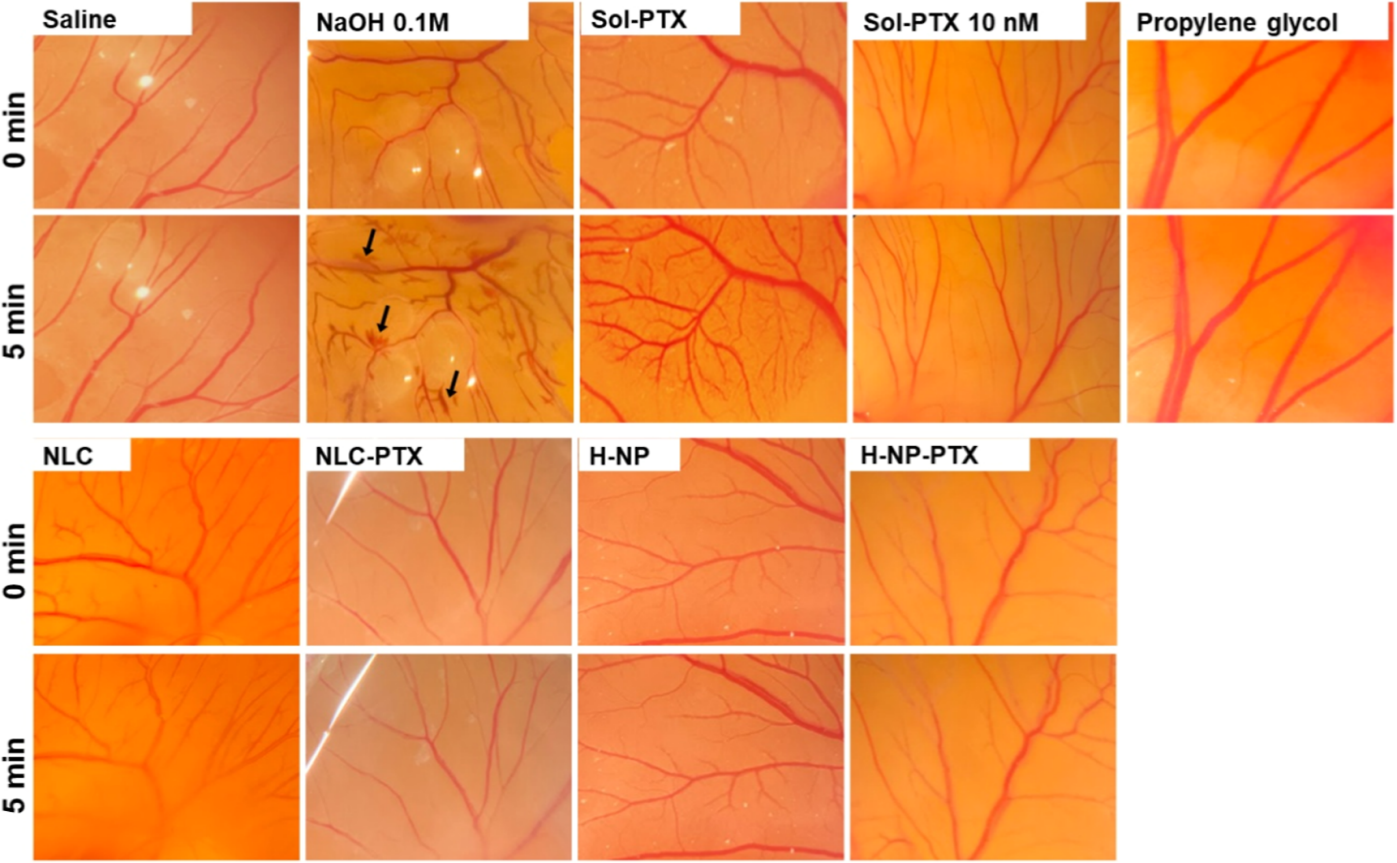
Assessment of irritation potential on the chorioallantoic membrane of eggs. Signs of hemorrhage, coagulation, and vascular lysis were evaluated before treatment and 5 min of treatment with 0.1 M NaOH (positive control), saline (negative control), paclitaxel solution (1% in propylene glycol or diluted in saline to a concentration equivalent to 10 nM paclitaxel), propylene glycol (drug solution vehicle control), NLC, NLC-PTX, H-NP, and H-NP-PTX. Representative images from 3 eggs.

Dilution of paclitaxel solution in saline to a final concentration of 10 nM (a dose reported in the literature for antiangiogenic studies)^20^ did not lead to vascular events, suggesting that this concentration can be employed to assess its antiangiogenic effects.

#### 3.3.1 PTX Nanoencapsulation Did Not Prevent Its Antiangiogenic Effects, Regardless of the Type of Nanoparticle

Having demonstrated the safety of the nanoparticles in the chorioallantoic membrane, we next assessed the antiangiogenic effect of paclitaxel in solution or encapsulated in nanoparticles (Figure 6).

**Figure 6:**
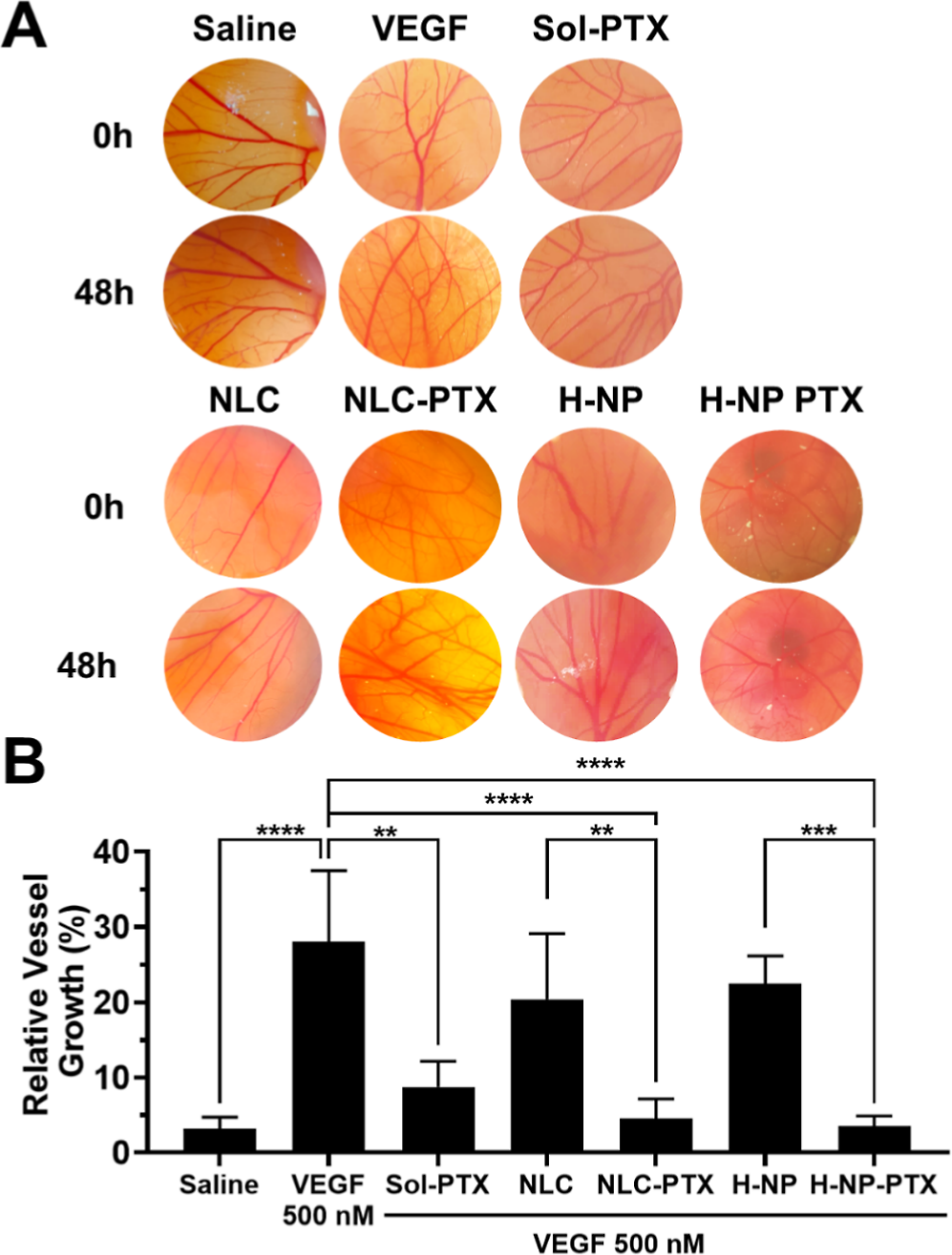
Evaluation of the antiangiogenic effect of paclitaxel on the chorioallantoic membrane of eggs. (A) Representative images of vessel growth and (B) relative vessel growth in eggs treated with saline (negative control), VEGF 500 nM (positive control), Sol-PTX (diluted in saline to an equivalent of 10 nM paclitaxel), NLC-PTX (diluted in saline to an equivalent of 10 nM paclitaxel), NLC (diluted in saline in the same proportion as NLC-PTX), H-NP-PTX (diluted in saline to an equivalent of 10 nM paclitaxel), H-NP (diluted in saline in the same proportion as H-NP-PTX). **p < 0.01, ***p < 0.001, and ****p < 0.0001.

Chorioallantoic membranes exposed to saline for 48 h exhibited a baseline blood vessel growth of 3.2% relative to time zero. In contrast, VEGF treatment resulted in a 28.1% increase in relative vascular growth. Treatment with paclitaxel, whether free or nanoencapsulated, significantly (*p* < 0.0001) reduced blood vessel formation by 3.3–7.8-fold compared to the VEGF group; relative growth was 8.7%, 4.6%, and 3.6% for Sol-PTX, NLC-PTX, and H-NP-PTX, respectively. Unloaded nanoparticles (NLC and H-NP) produced a modest reduction in vessel formation (1.2–1.3-fold), suggesting that the antiangiogenic effect is mediated primarily by paclitaxel.

## 4 Discussion

Nanotechnology-based drug delivery systems have emerged as a promising strategy to overcome the limitations of conventional paclitaxel formulations, such as off-target toxicity and limited bioavailability.^31^ In our previous studies, we characterized nanostructured lipid carriers and lipid-polymeric hybrid nanoparticles for paclitaxel delivery and demonstrated in vitro cytotoxic effects against breast cancer cells, with few differences observed depending on the nanocarrier type.^13,14^ Despite these similarities, these systems present distinct architectures and properties, particularly with respect to the presence of a polymeric shell functionalized with a collagen-binding peptide and drug release kinetics,^13^ which might differentially modulate paclitaxel availability and pharmacodynamics. Building upon these differences, this study aimed to investigate whether these nanocarriers differentially influence the molecular, irritant, and antiangiogenic effects of paclitaxel.

We initiated this study by analyzing the molecular effects of paclitaxel, either free or nanoencapsulated, focusing on changes in the expression of proteins related to microtubule stabilization, apoptosis, and DNA damage, which are commonly altered after treatment with paclitaxel.^28,32–34^ The expression of BAX, which provides an estimation of pro-apoptotic signaling,^28,35^ was pronouncedly increased after treatment with NLC-PTX and H-NP-PTX. Cleaved PARP-1 was detected in cells treated with both free and nanoencapsulated paclitaxel, particularly in MDA-MB-231 cells treated with NLC-PTX. PARP-1 cleavage is a known marker of apoptosis and has been previously observed in PTX-treated cells.^36^ These findings suggest that paclitaxel-loaded nanoparticles promoted proapoptotic signaling and that the type of nanoparticle did not interfere with the main mechanism of cell death induced by the drug.

Both free and nanoencapsulated paclitaxel increased the acetylation of α-tubulin in both cell lines, indicating that nanoencapsulation did not impair the drug’s microtubule-stabilizing mechanism. However, paclitaxel-loaded hybrid nanoparticles showed a reduced microtubule-stabilizing effect compared with free paclitaxel or NLC-PTX. We hypothesized that this apparent discrepancy might be attributed to differences in drug release kinetics between the formulations: paclitaxel release from H-NPs is slower than that from NLCs, probably because of the presence of multiple diffusion barriers imposed by the polymeric shell and surface functionalization, with only approximately 50% of the encapsulated drug released after 48 h.^13^ This slower release profile might result in reduced intracellular drug availability during early time points, leading to diminished tubulin binding relative to free paclitaxel or paclitaxel-loaded NLC.

Interestingly, unloaded nanoparticles induced a modest but detectable increase in tubulin acetylation, suggesting that the nanocarrier itself may interfere with the cytoskeletal dynamics. This biological effect was supported by immunofluorescence analysis, which revealed morphological changes consistent with mitotic arrest following treatment with both drug-loaded and unloaded nanoparticles. Similar nanoparticle–tubulin interactions have been described for gold nanoparticles,^37^ underscoring the importance of evaluating the intrinsic biological activity of nanocarriers. These findings were interpreted as potentially beneficial considering localized drug delivery through the proposed intraductal route and prolonged retention within the mammary tissue, which may limit systemic exposure and reduce adverse effects in normal tissues. This potential benefit becomes more apparent if we consider surface modification of the hybrid nanoparticles to improve selectivity: in a previous study from our group, we demonstrated that their surface modification with the collagen-binding peptide SILY reduced the viability of nontumor breast cells in a less pronounced manner compared to cancer cells in a coculture model, supporting selectivity improvement.^13^

Nanomaterials may induce cytotoxicity through multiple mechanisms, ranging from direct DNA damage and other intracellular signaling pathways associated with cell death.^10,11^ These effects are highly dependent on composition and physicochemical properties such as size, surface charge, and interaction with biological membranes.^12^ Thus, biological effects may arise either from the intrinsic activity of formulation components or from interactions between the lipid nanostructure and cellular membranes. For example, liposome-encapsulated doxorubicin (Lipodox) has shown enhanced efficacy in drug-resistant cancer cells compared to free doxorubicin,^13^ attributed not only to drug delivery but also to the ability of the liposomal shell itself to alter membrane lipid raft composition, reducing the localization and activity of *P*-glycoprotein (Pgp), a key efflux transporter associated with multidrug resistance. A different study demonstrated that lipid composition is an important determinant of nanoparticle cytotoxicity. For example, nanoparticles composed of branched lipids, such as phytantriol, exhibited significantly higher toxicity than those containing unsaturated lipids, such as monoolein.^14^ We hypothesized that tributyrin, present in both NLC and H-NP formulations, may contribute to this effect. Wang et al. demonstrated that tributyrin can increase tubulin acetylation in endothelial cells through inhibition of histone deacetylase 6 (HDAC6), the enzyme responsible for tubulin deacetylation.^38^ However, this mechanism was not directly evaluated in this study and therefore remains speculative. In the context of our study, the most relevant aspect is that tubulin acetylation, independently of direct HDAC6 activity, is widely recognized as a functional biomarker of paclitaxel activity and that this effect may be enhanced by the nanocarrier. Indeed, paclitaxel-induced microtubule stabilization is closely associated with increased acetylation of α-tubulin (particularly at lysine 40), which serves as a marker of stable, long-lived microtubules.^39^

In addition to cytoskeletal modulation, γH2AX levels (a marker of DNA double-strand breaks) were substantially increased in cells treated with PTX-loaded nanocarriers but not paclitaxel in solution. This suggests that nanoencapsulation may potentiate PTX-induced DNA damage,^27^ possibly by enhancing cellular uptake or nanoparticle components’ effects. This effect was more pronounced for NLC-PTX compared with H-NP-PTX or Sol-PTX in both cell lines, which may correlate with the greater cytotoxicity observed for the lipid-based carrier in our previous studies.^13^ We speculate that tributyrin may also contribute to this effect, as histone deacetylase inhibitors have been associated with suppression of DNA double-strand break repair in breast cancer cells,^40^ but this mechanism was not investigated here. Finally, although there is no direct evidence in the literature demonstrating that SILY modulates the specific signaling pathways investigated in this study, its function as a collagen-targeting moiety may indirectly influence cellular responses. By enhancing nanoparticle retention within collagen-rich extracellular matrix regions, SILY may increase local drug exposure and prolong nanoparticle–cell interactions, potentially contributing to other downstream molecular effects.^7,8^ Nevertheless, further studies are required to elucidate the direct involvement of SILY in these signaling events.

Altogether, these findings highlight the potential of nanocarriers not only to effectively deliver paclitaxel but also to modulate and enhance its antitumor mechanisms. Moreover, the stronger effects observed for NLCs compared with those for hybrid nanoparticles on DNA damage marker expression and cytoskeletal modulation support the greater cytotoxic activity previously observed for this system and demonstrate that the type of nanocarrier can influence the pharmacodynamic effects.

We also investigated the antiangiogenic properties of paclitaxel either in solution or nanoencapsulated. The antiangiogenic activity of chemotherapeutic agents has been described under both conventional (high-dose, cyclic) and metronomic (low-dose, continuous) regimens.^41,42^ Metronomic chemotherapy, in particular, relies on sustained drug exposure and has gained interest for its ability to suppress neovascularization at subcytotoxic concentrations while reducing systemic toxicity.^43,44^ In this context, the nanoparticle formulations developed in this study were designed to provide prolonged drug release and may act as a potential platform for metronomic delivery of paclitaxel, enhancing its antiangiogenic efficacy while minimizing adverse effects.

Previous studies have demonstrated the antiangiogenic effect of paclitaxel in preclinical breast cancer models.^45,46^ For instance, Jiang et al. compared daily low-dose (1.3 mg/kg) PTX with a high-dose weekly regimen (20 mg/kg) in a 4T1 metastatic breast cancer model.^45^ The metronomic treatment significantly reduced tumor volume (by 2.2-fold) and pulmonary metastases (by 1.8-fold) with fewer adverse effects. Additionally, the metronomic dose resulted in more pronounced angiogenesis inhibition, as made evident by immunohistochemistry staining for CD31. Similarly, Hsu et al. reported that sustained release of PTX by subcutaneously implanted PLGA nanofibers (low dose of 1,2 mg/rat) reduced tumor volume and angiogenesis more effectively than conventional dosing in an orthotopic MDA-MB-231 xenograft model.^46^

The antiangiogenic effects of paclitaxel were evaluated using a chorioallantoic membrane (CAM) assay. A reduced number of embryos was used in this study in strict accordance with the principles of the 3Rs (reduction, refinement, and replacement), aiming to minimize embryo use while maintaining scientific validity. We acknowledge that this limited sample size may reduce the statistical power and, thus, represents a limitation of the present study. Paclitaxel was tested at a subcytotoxic concentration (10 nM), in alignment with metronomic dosing strategies. Treatments with paclitaxel in solution, NLC-PTX, and H-NP-PTX effectively inhibited blood vessel formation, reducing vascular growth by 3.2-, 6.1-, and 7.8-fold, respectively. The slightly more pronounced antiangiogenic effect observed for H-NP-PTX may be related not only to sustained drug release, which aligns with the principles of metronomic chemotherapy, but also to the presence of the targeting SILY peptide, which may favor interactions with endothelial cells.^47^ Importantly, the NLC and H-NPs alone did not alter angiogenesis compared to the VEGF control, suggesting that these carriers did not interfere with normal angiogenic processes and may therefore not affect healthy cells to an appreciable degree.

## 5 Conclusions

In this study, we investigated the effects of paclitaxel nanoencapsulation using two distinct systems, nanostructured lipid carriers (NLCs) and lipid-polymeric hybrid nanoparticles (H-NPs)—on key mechanisms associated with its antitumor activity. Both nanocarriers preserved paclitaxel’s ability to stabilize microtubules and induce apoptosis. Comparative analysis indicated that NLCs exerted more pronounced effects on DNA damage and cytoskeletal modulation than H-NPs, especially in MCF-7 cells, suggesting that the type or specific components of carriers may potentiate paclitaxel’s effects. Importantly, both PTX-loaded NLC and H-NP maintained the drug antiangiogenic activity, significantly reducing blood vessel formation in vivo. In contrast, unloaded NLCs and H-NPs did not alter angiogenesis, indicating that the observed effects were primarily drug-driven. Collectively, these findings demonstrate that nanoencapsulation can modulate paclitaxel’s biological effects in a carrier-dependent manner and highlight the potential of NLCs and H-NPs as promising delivery platforms for breast cancer therapy.

## Supplementary Material



## Notes

List of antibodies used, their characteristics, and associated cellular processes (PDF)

## References

[1] Abu
Samaan T. M., Samec M., Liskova A., Kubatka P., Büsselberg D. (2019). Paclitaxel’s Mechanistic and Clinical Effects
on Breast Cancer. Biomolecules.

[2] Burguin A., Diorio C., Durocher F. (2021). Breast Cancer Treatments: Updates
and New Challenges. J. Pers. Med..

[3] Lim, P. T. ; Goh, B. H. ; Lee, W. 3 - Taxol: Mechanisms of action against cancer, an update with current research. In Paclitaxel; Swamy, M. K. , Pullaiah, T. , Chen, Z. , Eds.; Academic Press, 2022; pp 47–71.

[4] Kampan N. C., Madondo M. T., McNally O. M., Quinn M., Plebanski M. (2015). Paclitaxel
and Its Evolving Role in the Management of Ovarian Cancer. BioMed Res. Int..

[5] Khing T. M., Choi W. S., Kim D. M., Po W. W., Thein W., Shin C. Y., Sohn U. D. (2021). The effect
of paclitaxel on apoptosis,
autophagy and mitotic catastrophe in AGS cells. Sci. Rep..

[6] Bocci G., Di Paolo A., Danesi R. (2013). The pharmacological
bases of the
antiangiogenic activity of paclitaxel. Angiogenesis.

[7] Gelderblom H. (2001). Cremophor EL: the drawbacks
and advantages of vehicle selection for
drug formulation. Eur. J. Cancer.

[8] Miele E. (2009). Albumin-bound formulation
of paclitaxel (Abraxane ABI-007) in the
treatment of breast cancer. Int. J. Nanomed..

[9] Mohammad
Jafari R., Ala M., Goodarzi N., Dehpour A. R. (2020). Does Pharmacodynamics
of Drugs Change After Presenting them as Nanoparticles Like their
Pharmacokinetics?. Curr. Drug Targets.

[10] Haripriyaa M., Suthindhiran K. (2023). Pharmacokinetics
of nanoparticles: current knowledge,
future directions and its implications in drug delivery. Future J. Pharmaceut. Sci..

[11] Chen L., Wu L. Y., Yang W. X. (2018). Nanoparticles induce
apoptosis via
mediating diverse cellular pathways. Nanomedicine.

[12] de Jesus, M. B. ; Kapila, Y. L. Cellular Mechanisms in Nanomaterial Internalization, Intracellular Trafficking, and Toxicity. In Nanotoxicology. Nanomedicine and Nanotoxicology; Durán, N. , Guterres, S. , Alves, O. , Eds.; Springer, 2014.

[13] Passos J.
S., Lopes L. B., Panitch A. (2023). Collagen-Binding Nanoparticles for
Paclitaxel Encapsulation and Breast Cancer Treatment. ACS Biomater. Sci. Eng..

[14] Passos J. S. (2024). Nanostructured lipid
carriers loaded into in situ gels for breast
cancer local treatment. Eur. J. Pharm. Sci..

[15] Sapienza
Passos J. (2023). Contributions of nanotechnology to the intraductal
drug delivery for local treatment and prevention of breast cancer. Int. J. Pharm..

[16] McFadden M., Singh S. K., Kinnel B., Varambally S., Singh R. (2023). The effect of paclitaxel- and fisetin-loaded
PBM nanoparticles on
apoptosis and reversal of drug resistance gene ABCG2 in ovarian cancer. J. Ovarian Res..

[17] Lima K., Carvalho M. F. L., Pereira-Martins D. A., Nogueira F. L., de Miranda L. B. L., Nascimento M. C. d., Cavaglieri R. d. C., Schuringa J. J., Machado-Neto J. A., Rego E. M. (2023). Pharmacological
Inhibition of PIP4K2 Potentiates Venetoclax-Induced Apoptosis in Acute
Myeloid Leukemia. Int. J. Mol. Sci..

[18] Fukumori C. (2025). Polymer-lipid hybrid microcarriers for oral
codelivery of paclitaxel
and tributyrin: development, optimization, and cytotoxicity in cells
and spheroids of colorectal cancer. Int. J.
Pharm..

[19] Hirokawa C. M. (2024). Evaluation of polyelectrolyte
nanoparticles of chitosan and hyaluronic
acid as topical delivery systems for cytotoxic agents. Colloids Surf., A.

[20] Vacca A. (2002). Docetaxel versus paclitaxel
for antiangiogenesis. J. Hematother. Stem Cell
Res..

[21] ICCVAM ICCVAM Test Method Evaluation Report: Current Validation Status of in Vitro Test Methods Proposed for Identifying Eye Injury Hazard Potential of Chemicals and Products; NIH Publication No, 2010; Vol. 10, p 7553.

[22] Wilting J. (1993). In vivo effects of vascular endothelial growth factor on the chicken
chorioallantoic membrane. Cell Tissue Res..

[23] Taktak-BenAmar A., Morjen M., Ben Mabrouk H., Abdelmaksoud-Dammak R., Guerfali M., Fourati-Masmoudi N., Marrakchi N., Gargouri A. (2017). Expression, purification and functionality
of bioactive
recombinant human vascular endothelial growth factor VEGF(165) in
E. coli. AMB Express.

[24] Bai Y. (2014). Effects of combinations of BMP-2 with FGF-2 and/or
VEGF on HUVECs
angiogenesis in vitro and CAM angiogenesis in vivo. Cell Tissue Res..

[25] Dallemole D. R., Terroso T., Alves A. d. C. S., Scholl J. N., Onzi G. R., Cé R., Paese K., Battastini A. M. O., Guterres S. S., Figueiró F. (2021). Nanoformulation Shows
Cytotoxicity against Glioblastoma Cell Lines and Antiangiogenic Activity
in Chicken Chorioallantoic Membrane. Pharmaceutics.

[26] Howes S. C. (2014). Effects of tubulin acetylation
and tubulin acetyltransferase binding
on microtubule structure. Mol. Biol. Cell.

[27] Kuo L. J., Yang L. X. (2008). Gamma-H2AX - a novel
biomarker for DNA double-strand
breaks. Vivo.

[28] Edlich F. (2018). BCL-2 proteins
and apoptosis: Recent insights and unknowns. Biochem. Biophys. Res. Commun..

[29] Weaver A. N., Yang E. S. (2013). Beyond DNA Repair: Additional Functions
of PARP-1 in
Cancer. Front. Oncol..

[30] Mascaraque M., Delgado-Wicke P., Damian A., Lucena S., Carrasco E., Juarranz Á. (2019). Mitotic
Catastrophe Induced in HeLa Tumor Cells by
Photodynamic Therapy with Methyl-aminolevulinate. Int. J. Mol. Sci..

[31] Ma P., Mumper R. J. (2013). Paclitaxel Nano-Delivery
Systems: A Comprehensive Review. J. Nanomed.
Nanotechnol..

[32] Alalawy A. I. (2024). Key genes
and molecular mechanisms related to Paclitaxel Resistance. Cancer Cell Int..

[33] Alqahtani F. Y. (2019). Chapter Three - Paclitaxel. Profiles Drug Subst.
Excipients Relat. Methodol..

[34] Weaver B. A. (2014). How Taxol/paclitaxel
kills cancer cells. Mol. Biol. Cell.

[35] Salata G. C. (2025). Molecular effects of
paclitaxel-elacridar nanoemulsions in breast
cancer cells: impact on uptake, cell cycle and signaling pathways. Eur. J. Pharm. Biopharm..

[36] Xiao W. Y. (2019). Paclitaxel Induce Apoptosis
of Giant Cells Tumor of Bone via TP53INP1
Signaling. Orthop. Surg..

[37] Mahaddalkar T. (2017). Tryptone-stabilized gold nanoparticles target tubulin and inhibit
cell viability by inducing an unusual form of cell cycle arrest. Exp. Cell Res..

[38] Wang Y. (2010). Normal Shear Stress and Vascular Smooth Muscle Cells
Modulate Migration
of Endothelial Cells Through Histone Deacetylase 6 Activation and
Tubulin Acetylation. Ann. Biomed. Eng..

[39] Janke C., Montagnac G. (2017). Causes and
Consequences of Microtubule Acetylation. Curr.
Biol..

[40] Li L., Sun Y., Liu J., Wu X., Chen L., Ma L., Wu P. (2015). Histone deacetylase
inhibitor sodium butyrate suppresses DNA double
strand break repair induced by etoposide more effectively in MCF-7
cells than in HEK293 cells. BMC Biochem..

[41] Liu Z. L., Chen H. H., Zheng L. L., Sun L. P., Shi L. (2023). Angiogenic
signaling pathways and anti-angiogenic therapy for cancer. Signal Transduct. Targeted Ther..

[42] Munoz R., Girotti A., Hileeto D., Arias F. J. (2021). Metronomic
Anti-Cancer
Therapy: A Multimodal Therapy Governed by the Tumor Microenvironment. Cancers.

[43] Kirti A. (2024). Nanoparticle-mediated
metronomic chemotherapy in cancer: A paradigm
of precision and persistence. Cancer Lett..

[44] Panthi V. K., Dua K., Singh S. K., Gupta G., Hansbro P. M., Paudel K. R. (2023). Nanoformulations-Based
Metronomic Chemotherapy: Mechanism, Challenges, Recent Advances, and
Future Perspectives. Pharmaceutics.

[45] Jiang H. (2010). Low-dose metronomic paclitaxel chemotherapy suppresses
breast tumors
and metastases in mice. Cancer Invest..

[46] Hsu M. Y., Hsieh C. H., Huang Y. T., Chu S. Y., Chen C. M., Lee W. J., Liu S. J. (2021). Enhanced
Paclitaxel Efficacy to Suppress
Triple-Negative Breast Cancer Progression Using Metronomic Chemotherapy
with a Controlled Release System of Electrospun Poly-d-l-Lactide-Co-Glycolide
(PLGA) Nanofibers. Cancers.

[47] McMasters J., Panitch A. (2017). Collagen-binding nanoparticles for extracellular anti-inflammatory
peptide delivery decrease platelet activation, promote endothelial
migration, and suppress inflammation. Acta Biomater..

