# Supplementary material for: Molecular and Antiangiogenic Effects of Paclitaxel-Loaded Nanoparticles: Influence of the Nanocarrier Type
Source: Mol Pharm. 2026 Mar 6;23(4):2626–37. doi: 10.1021/acs.molpharmaceut.5c01740 (PMC13058873; doi:10.1021/acs.molpharmaceut.5c01740)
Supplement: Supplementary file 1 [file mp5c01740_si_001.pdf]

Supporting Information

Molecular and Antiangiogenic Effects of Paclitaxel-Loaded Nanoparticles: Influence of the Nanocarrier Type

Julia Sapienza Passos [1], Giovanna B. de Melo [1], Giovanna C. Salata [1], João Agostinho Machado-Neto [1], Alyssa Panitch [2], Luciana B. Lopes [1*]

[1] Department of Pharmacology, Institute of Biomedical Sciences, University of Sao Paulo, Sao Paulo, SP 05508-000, Brazil

[2] Wallace H. Coulter Department of Biomedical Engineering, Georgia Institute of Technology and Emory University, Atlanta, Georgia 30332, United States

Table of contents

This document contains one table depicting supporting results, as listed below.

Table S1. List of antibodies used, their characteristics, and associated cellular processes.

Table S1. List of antibodies used, their characteristics, and associated cellular processes.

Antibody	Company	Cellular Process	Molecular Weight	Isotype	Dilution
Anti-rat	Cell Signaling	Secondary antibody	-	Goat	1:2000
α-tubulin	Cell Signaling	Loading control	50 kDa	Mouse	1:4000
Acetyl-α-tubulin	Cell Signaling	Microtubule stability	52 kDa	Mouse	1:4000
BAX	Invitrogen	Apoptosis	21 kDa	Mouse	1:2000
γH2AX	Cell Signaling	DNA damage	15 kDa	Mouse	1:4000
PARP1	Cell Signaling	Apoptosis	116 kDa, 89 kDa (cleaved)	Mouse	1:2000
